# Supplementary material for: Proteolytic processing of palmitoylated Hedgehog peptides specifies the 3-4 intervein region of the Drosophila wing
Source: eLife. 2018 Mar 9;7:e33033. doi: 10.7554/eLife.33033 (PMC5844694; doi:10.7554/eLife.33033)
Supplement: Supplementary file 1. [file elife-33033-supp1.docx]

Supplementary file 1

Mutagenesis primers used for the consecutive generation of

Hh^C85S^ 1: 5‘CAATCCTCGGCCAGGACCGCTGCTGTGAGCCGGGCTAAAGAC3‘

2: 5‘GTCTTTAGCCCGGCTCACAGCAGCGGTCCTGGCCGAGGATTG3‘

Hh^C85S;Δ86-91^  1: 5’TTTAGCCCGGCTCACAGCAGCGGTCGTCATAGGGCGCGCAAC3’

2: 5‘GTTGCGCGCCCTATGACGACCGCTGCTGTGAGCCGGGCTAAA3‘

Hh^C85S;Δ86-92^ 1: 5‘TTTAGCCCGGCTCACAGCAGCCGTCATAGGGCGCGCAACCTG3‘

2: 5‘CAGGTTGCGCGCCCTATGACGGCTGCTGTGAGCCGGGCTAAA3‘

Hh^C85S;Δ86-93^ 1: 5‘ATACAGGTTGCGCGCCCTATGGCTGCTGTGAGCCGGGCTAAA3‘

2: 5‘TTTAGCCCGGCTCACAGCAGCCATAGGGCGCGCAACCTGTAT3‘

Hh^C85S;Δ86-94^ 1: 5’TTTAGCCCGGCTCACAGCAGCAGGGCGCGCAACCTGTATCC3‘

2: 5‘GGATACAGGTTGCGCGCCCTGCTGCTGTGAGCCGGGCTAAA3‘

Hh^C85S;Δ86-95^ 1: 5‘TTTAGCCCGGCTCACAGCAGCGCGCGCAACCTGTATCCGCTG3‘

2: 5‘CAGCGGATACAGGTTGCGCGCGCTGCTGTGAGCCGGGCTAAA3‘

Hh^C85S;Δ86-96^ 1: 5‘TTTAGCCCGGCTCACAGCAGCCGCAACCTGTATCCGCTGGTC3‘

2: 5’GACCAGCGGATACAGGTTGCGGCTGCTGTGAGCCGGGCTAAA3’

Hh^C85S;Δ86-97^ 1: 5‘TTTAGCCCGGCTCACAGCAGCAACCTGTATCCGCTGGTCCTC3‘

2: 5’GAGGACCAGCGGATACAGGTTGCTGCTGTGAGCCGGGCTAAA3’

Hh^C85S;Δ86-98^ 1: 5‘TTTAGCCCGGCTCACAGCAGCCTGTATCCGCTGGTCCTCAAG3‘

2: 5’CTTGAGGACCAGCGGATACAGGCTGCTGTGAGCCGGGCTAAA3’

Hh^C85S;Δ86-99^ 1: 5‘CTGCTTGAGGACCAGCGGATAGCTGCTGTGAGCCGGGCTAAA3’

2: 5’TTTAGCCCGGCTCACAGCAGCTATCCGCTGGTCCTCAAGCAG3’

Hh^C85S;Δ86-100^ 1: 5‘CACCATGGATAACCACAGCTCAGTG3‘

2: 5‘TCAATCGTGGCGCCAGCTC3‘

^HA^Hh^C85S^ 1: 5‘GCGGTCCTGGCCGAGGATTGTACCCATACGATGTTCCAGATTACGCTGGTCGTCATAGGGCGCGCAAC3‘

2: 5‘GTTGCGCGCCCTATGACGACCAGCGTAATCTGGAACATCGTATGGGTACAATCCTCGGCCAGGACCGC3‘

^HA^Hh 1: 5‘GCGGTCCTGGCCGAGGATTGTACCCATACGATGTTCCAGATTACGCTGGTCGTCATAGGGCGCGCAAC3‘

2: 5‘GTTGCGCGCCCTATGACGACCAGCGTAATCTGGAACATCGTATGGGTACAATCCTCGGCCAGGACCGC3‘

HhN^C85S^ 1: 5‘CACCATGGATAACCACAGCTCAGTG3‘

2: 5‘GCACTAGTGCACGTGGGAA3‘

Shh 1: 5‘AACTCGAGATGCTGCTGCTGCTGGCC3‘

2: 5‘AAACGCGTTCAGCTGGACTTGACCGCC3‘

Sequence confirmation of constructs used for the generation of transgenic flies

Dm Hedgehog: NM_001038976.1

atggataaccacagctcagtgccttgggccagtgccgccagtgtcacctgtctctccctggatgccaaatgccacagttccagttccagttccagctccaaatccgcagcgagctccatctccgcaatcccgcaagaagaaacgcaaacgatgcgccacattgcgcatacgcagcgttgcctcagcaggctgacctctctggtggccctgctgctgatcgtcttgccgatggtctttagcccggctcacagctgcggtcctggccgaggattgggtcgtcatagggcgcgcaacctgtatccgctggtcctcaagcagacaattcccaatctatccgagtacacgaacagcgcctccggacctctggagggtgtgatccgtcgggactcgcccaaattcaaggacctcgtgcccaactacaacagggacatccttttccgcgacgaggaaggcaccggagcggatcgcttgatgagcaagcgctgcaaggagaagctaaacgtgctggcctactcggtgatgaacgaatggcccggcatccggctgctggtcaccgagagctgggacgaggactaccatcacggccaggagtcgctccactacgagggccgagcggtgaccattgccacctccgatcgcgaccagtccaaatacggcatgctcgctcgcctggccgtcgaggctggattcgattgggtctcctacgtcagcaggcgccacatctactgctccgtcaagtcagattcgtcgatcagttcccacgtgcacggctgcttcacgccggagagcacagcgctgctggagagtggagtccggaagccgctcggcgagctctctatcggagatcgtgttttgagcatgaccgccaacggacaggccgtctacagcgaagtgatcctcttcatggaccgcaacctcgagcagatgcaaaactttgtgcagctgcacacggacggtggagcagtgctcacggtgacgccggctcacctggttagcgtttggcagccggagagccagaagctcacgtttgtgtttgcggatcgcatcgaggagaagaaccaggtgctcgtacgggatgtggagacgggcgagctgaggccccagcgagtcgtcaaggtgggcagtgtgcgcagtaagggcgtggtcgcgccgctgacccgcgagggcaccattgtggtcaactcggtggccgccagttgctatgcggtgatcaacagccagtcgctggcccactggggactggctcccatgcgcctgctgtccacgctggaggcgtggctgcccgccaaggagcagttgcacagttcgccgaaggtggtgagctcggcgcagcagcagaatggcatccattggtatgccaatgcgctctacaaggtcaaggactacgttctgccgcagagctggcgccacgat

MDNHSSVPWASAASVTCLSLDAKCHSSSSSSSSKSAASSISAIPQEETQTMRHIAHTQRCLSRLTSLVALLLIVLPMVFSPAHSCGPGRGLGRHRARNLYPLVLKQTIPNLSEYTNSASGPLEGVIRRDSPKFKDLVPNYNRDILFRDEEGTGADRLMSKRCKEKLNVLAYSVMNEWPGIRLLVTESWDEDYHHGQESLHYEGRAVTIATSDRDQSKYGMLARLAVEAGFDWVSYVSRRHIYCSVKSDSSISSHVHGCFTPESTALLESGVRKPLGELSIGDRVLSMTANGQAVYSEVILFMDRNLEQMQNFVQLHTDGGAVLTVTPAHLVSVWQPESQKLTFVFADRIEEKNQVLVRDVETGELRPQRVVKVGSVRSKGVVAPLTREGTIVVNSVAASCYAVINSQSLAHWGLAPMRLLSTLEAWLPAKEQLHSSPKVVSSAQQQNGIHWYANALYKVKDYVLPQSWRHD

Hh^C85S^

atggataaccacagctcagtgccttgggccagtgccgccagtgtcacctgtctctccctggatgccaaatgccacagttccagttccagttccagctccaaatccgcagcgagctccatctccgcaatcccgcaagaagaaacgcaaacgatgcgccacattgcgcatacgcagcgttgcctcagcaggctgacctctctggtggccctgctgctgatcgtcttgccgatggtctttagcccggctcacagcagcggtcctggccgaggattgggtcgtcatagggcgcgcaacctgtatccgctggtcctcaagcagacaattcccaatctatccgagtacacgaacagcgcctccggacctctggagggtgtgatccgtcgggactcgcccaaattcaaggacctcgtgcccaactacaacagggacatccttttccgcgacgaggaaggcaccggagcggatcgcttgatgagcaagcgctgcaaggagaagctaaacgtgctggcctactcggtgatgaacgaatggcccggcatccggctgctggtcaccgagagctgggacgaggactaccatcacggccaggagtcgctccactacgagggccgagcggtgaccattgccacctccgatcgcgaccagtccaaatacggcatgctcgctcgcctggccgtcgaggctggattcgattgggtctcctacgtcagcaggcgccacatctactgctccgtcaagtcagattcgtcgatcagttcccacgtgcacggctgcttcacgccggagagcacagcgctgctggagagtggagtccggaagccgctcggcgagctctctatcggagatcgtgttttgagcatgaccgccaacggacaggccgtctacagcgaagtgatcctcttcatggaccgcaacctcgagcagatgcaaaactttgtgcagctgcacacggacggtggagcagtgctcacggtgacgccggctcacctggttagcgtttggcagccggagagccagaagctcacgtttgtgtttgcggatcgcatcgaggagaagaaccaggtgctcgtacgggatgtggagacgggcgagctgaggccccagcgagtcgtcaaggtgggcagtgtgcgcagtaagggcgtggtcgcgccgctgacccgcgagggcaccattgtggtcaactcggtggccgccagttgctatgcggtgatcaacagccagtcgctggcccactggggactggctcccatgcgcctgctgtccacgctggaggcgtggctgcccgccaaggagcagttgcacagttcgccgaaggtggtgagctcggcgcagcagcagaatggcatccattggtatgccaatgcgctctacaaggtcaaggactacgttctgccgcagagctggcgccacgat

MDNHSSVPWASAASVTCLSLDAKCHSSSSSSSSKSAASSISAIPQEETQTMRHIAHTQRCLSRLTSLVALLLIVLPMVFSPAHSSGPGRGLGRHRARNLYPLVLKQTIPNLSEYTNSASGPLEGVIRRDSPKFKDLVPNYNRDILFRDEEGTGADRLMSKRCKEKLNVLAYSVMNEWPGIRLLVTESWDEDYHHGQESLHYEGRAVTIATSDRDQSKYGMLARLAVEAGFDWVSYVSRRHIYCSVKSDSSISSHVHGCFTPESTALLESGVRKPLGELSIGDRVLSMTANGQAVYSEVILFMDRNLEQMQNFVQLHTDGGAVLTVTPAHLVSVWQPESQKLTFVFADRIEEKNQVLVRDVETGELRPQRVVKVGSVRSKGVVAPLTREGTIVVNSVAASCYAVINSQSLAHWGLAPMRLLSTLEAWLPAKEQLHSSPKVVSSAQQQNGIHWYANALYKVKDYVLPQSWRHD

Hh^C85S;Δ86-91^

atggataaccacagctcagtgccttgggccagtgccgccagtgtcacctgtctctccctggatgccaaatgccacagttccagttccagttccagctccaaatccgcagcgagctccatctccgcaatcccgcaagaagaaacgcaaacgatgcgccacattgcgcatacgcagcgttgcctcagcaggctgacctctctggtggccctgctgctgatcgtcttgccgatggtctttagcccggctcacagcagcggtcgtcatagggcgcgcaacctgtatccgctggtcctcaagcagacaattcccaatctatccgagtacacgaacagcgcctccggacctctggagggtgtgatccgtcgggactcgcccaaattcaaggacctcgtgcccaactacaacagggacatccttttccgcgacgaggaaggcaccggagcggatcgcttgatgagcaagcgctgcaaggagaagctaaacgtgctggcctactcggtgatgaacgaatggcccggcatccggctgctggtcaccgagagctgggacgaggactaccatcacggccaggagtcgctccactacgagggccgagcggtgaccattgccacctccgatcgcgaccagtccaaatacggcatgctcgctcgcctggccgtcgaggctggattcgattgggtctcctacgtcagcaggcgccacatctactgctccgtcaagtcagattcgtcgatcagttcccacgtgcacggctgcttcacgccggagagcacagcgctgctggagagtggagtccggaagccgctcggcgagctctctatcggagatcgtgttttgagcatgaccgccaacggacaggccgtctacagcgaagtgatcctcttcatggaccgcaacctcgagcagatgcaaaactttgtgcagctgcacacggacggtggagcagtgctcacggtgacgccggctcacctggttagcgtttggcagccggagagccagaagctcacgtttgtgtttgcggatcgcatcgaggagaagaaccaggtgctcgtacgggatgtggagacgggcgagctgaggccccagcgagtcgtcaaggtgggcagtgtgcgcagtaagggcgtggtcgcgccgctgacccgcgagggcaccattgtggtcaactcggtggccgccagttgctatgcggtgatcaacagccagtcgctggcccactggggactggctcccatgcgcctgctgtccacgctggaggcgtggctgcccgccaaggagcagttgcacagttcgccgaaggtggtgagctcggcgcagcagcagaatggcatccattggtatgccaatgcgctctacaaggtcaaggactacgttctgccgcagagctggcgccacgat

MDNHSSVPWASAASVTCLSLDAKCHSSSSSSSSKSAASSISAIPQEETQTMRHIAHTQRCLSRLTSLVALLLIVLPMVFSPAHSSGRHRARNLYPLVLKQTIPNLSEYTNSASGPLEGVIRRDSPKFKDLVPNYNRDILFRDEEGTGADRLMSKRCKEKLNVLAYSVMNEWPGIRLLVTESWDEDYHHGQESLHYEGRAVTIATSDRDQSKYGMLARLAVEAGFDWVSYVSRRHIYCSVKSDSSISSHVHGCFTPESTALLESGVRKPLGELSIGDRVLSMTANGQAVYSEVILFMDRNLEQMQNFVQLHTDGGAVLTVTPAHLVSVWQPESQKLTFVFADRIEEKNQVLVRDVETGELRPQRVVKVGSVRSKGVVAPLTREGTIVVNSVAASCYAVINSQSLAHWGLAPMRLLSTLEAWLPAKEQLHSSPKVVSSAQQQNGIHWYANALYKVKDYVLPQSWRHD

Hh^C85S;Δ86-92^

atggataaccacagctcagtgccttgggccagtgccgccagtgtcacctgtctctccctggatgccaaatgccacagttccagttccagttccagctccaaatccgcagcgagctccatctccgcaatcccgcaagaagaaacgcaaacgatgcgccacattgcgcatacgcagcgttgcctcagcaggctgacctctctggtggccctgctgctgatcgtcttgccgatggtctttagcccggctcacagcagccgtcatagggcgcgcaacctgtatccgctggtcctcaagcagacaattcccaatctatccgagtacacgaacagcgcctccggacctctggagggtgtgatccgtcgggactcgcccaaattcaaggacctcgtgcccaactacaacagggacatccttttccgcgacgaggaaggcaccggagcggatcgcttgatgagcaagcgctgcaaggagaagctaaacgtgctggcctactcggtgatgaacgaatggcccggcatccggctgctggtcaccgagagctgggacgaggactaccatcacggccaggagtcgctccactacgagggccgagcggtgaccattgccacctccgatcgcgaccagtccaaatacggcatgctcgctcgcctggccgtcgaggctggattcgattgggtctcctacgtcagcaggcgccacatctactgctccgtcaagtcagattcgtcgatcagttcccacgtgcacggctgcttcacgccggagagcacagcgctgctggagagtggagtccggaagccgctcggcgagctctctatcggagatcgtgttttgagcatgaccgccaacggacaggccgtctacagcgaagtgatcctcttcatggaccgcaacctcgagcagatgcaaaactttgtgcagctgcacacggacggtggagcagtgctcacggtgacgccggctcacctggttagcgtttggcagccggagagccagaagctcacgtttgtgtttgcggatcgcatcgaggagaagaaccaggtgctcgtacgggatgtggagacgggcgagctgaggccccagcgagtcgtcaaggtgggcagtgtgcgcagtaagggcgtggtcgcgccgctgacccgcgagggcaccattgtggtcaactcggtggccgccagttgctatgcggtgatcaacagccagtcgctggcccactggggactggctcccatgcgcctgctgtccacgctggaggcgtggctgcccgccaaggagcagttgcacagttcgccgaaggtggtgagctcggcgcagcagcagaatggcatccattggtatgccaatgcgctctacaaggtcaaggactacgttctgccgcagagctggcgccacgat

MDNHSSVPWASAASVTCLSLDAKCHSSSSSSSSKSAASSISAIPQEETQTMRHIAHTQRCLSRLTSLVALLLIVLPMVFSPAHSSRHRARNLYPLVLKQTIPNLSEYTNSASGPLEGVIRRDSPKFKDLVPNYNRDILFRDEEGTGADRLMSKRCKEKLNVLAYSVMNEWPGIRLLVTESWDEDYHHGQESLHYEGRAVTIATSDRDQSKYGMLARLAVEAGFDWVSYVSRRHIYCSVKSDSSISSHVHGCFTPESTALLESGVRKPLGELSIGDRVLSMTANGQAVYSEVILFMDRNLEQMQNFVQLHTDGGAVLTVTPAHLVSVWQPESQKLTFVFADRIEEKNQVLVRDVETGELRPQRVVKVGSVRSKGVVAPLTREGTIVVNSVAASCYAVINSQSLAHWGLAPMRLLSTLEAWLPAKEQLHSSPKVVSSAQQQNGIHWYANALYKVKDYVLPQSWRHD

Hh^C85S;Δ86-93^

atggataaccacagctcagtgccttgggccagtgccgccagtgtcacctgtctctccctggatgccaaatgccacagttccagttccagttccagctccaaatccgcagcgagctccatctccgcaatcccgcaagaagaaacgcaaacgatgcgccacattgcgcatacgcagcgttgcctcagcaggctgacctctctggtggccctgctgctgatcgtcttgccgatggtctttagcccggctcacagcagccatagggcgcgcaacctgtatccgctggtcctcaagcagacaattcccaatctatccgagtacacgaacagcgcctccggacctctggagggtgtgatccgtcgggactcgcccaaattcaaggacctcgtgcccaactacaacagggacatccttttccgcgacgaggaaggcaccggagcggatcgcttgatgagcaagcgctgcaaggagaagctaaacgtgctggcctactcggtgatgaacgaatggcccggcatccggctgctggtcaccgagagctgggacgaggactaccatcacggccaggagtcgctccactacgagggccgagcggtgaccattgccacctccgatcgcgaccagtccaaatacggcatgctcgctcgcctggccgtcgaggctggattcgattgggtctcctacgtcagcaggcgccacatctactgctccgtcaagtcagattcgtcgatcagttcccacgtgcacggctgcttcacgccggagagcacagcgctgctggagagtggagtccggaagccgctcggcgagctctctatcggagatcgtgttttgagcatgaccgccaacggacaggccgtctacagcgaagtgatcctcttcatggaccgcaacctcgagcagatgcaaaactttgtgcagctgcacacggacggtggagcagtgctcacggtgacgccggctcacctggttagcgtttggcagccggagagccagaagctcacgtttgtgtttgcggatcgcatcgaggagaagaaccaggtgctcgtacgggatgtggagacgggcgagctgaggccccagcgagtcgtcaaggtgggcagtgtgcgcagtaagggcgtggtcgcgccgctgacccgcgagggcaccattgtggtcaactcggtggccgccagttgctatgcggtgatcaacagccagtcgctggcccactggggactggctcccatgcgcctgctgtccacgctggaggcgtggctgcccgccaaggagcagttgcacagttcgccgaaggtggtgagctcggcgcagcagcagaatggcatccattggtatgccaatgcgctctacaaggtcaaggactacgttctgccgcagagctggcgccacgat

MDNHSSVPWASAASVTCLSLDAKCHSSSSSSSSKSAASSISAIPQEETQTMRHIAHTQRCLSRLTSLVALLLIVLPMVFSPAHSSHRARNLYPLVLKQTIPNLSEYTNSASGPLEGVIRRDSPKFKDLVPNYNRDILFRDEEGTGADRLMSKRCKEKLNVLAYSVMNEWPGIRLLVTESWDEDYHHGQESLHYEGRAVTIATSDRDQSKYGMLARLAVEAGFDWVSYVSRRHIYCSVKSDSSISSHVHGCFTPESTALLESGVRKPLGELSIGDRVLSMTANGQAVYSEVILFMDRNLEQMQNFVQLHTDGGAVLTVTPAHLVSVWQPESQKLTFVFADRIEEKNQVLVRDVETGELRPQRVVKVGSVRSKGVVAPLTREGTIVVNSVAASCYAVINSQSLAHWGLAPMRLLSTLEAWLPAKEQLHSSPKVVSSAQQQNGIHWYANALYKVKDYVLPQSWRHD

Hh^C85S;Δ86-94^

atggataaccacagctcagtgccttgggccagtgccgccagtgtcacctgtctctccctggatgccaaatgccacagttccagttccagttccagctccaaatccgcagcgagctccatctccgcaatcccgcaagaagaaacgcaaacgatgcgccacattgcgcatacgcagcgttgcctcagcaggctgacctctctggtggccctgctgctgatcgtcttgccgatggtctttagcccggctcacagcagcagggcgcgcaacctgtatccgctggtcctcaagcagacaattcccaatctatccgagtacacgaacagcgcctccggacctctggagggtgtgatccgtcgggactcgcccaaattcaaggacctcgtgcccaactacaacagggacatccttttccgcgacgaggaaggcaccggagcggatcgcttgatgagcaagcgctgcaaggagaagctaaacgtgctggcctactcggtgatgaacgaatggcccggcatccggctgctggtcaccgagagctgggacgaggactaccatcacggccaggagtcgctccactacgagggccgagcggtgaccattgccacctccgatcgcgaccagtccaaatacggcatgctcgctcgcctggccgtcgaggctggattcgattgggtctcctacgtcagcaggcgccacatctactgctccgtcaagtcagattcgtcgatcagttcccacgtgcacggctgcttcacgccggagagcacagcgctgctggagagtggagtccggaagccgctcggcgagctctctatcggagatcgtgttttgagcatgaccgccaacggacaggccgtctacagcgaagtgatcctcttcatggaccgcaacctcgagcagatgcaaaactttgtgcagctgcacacggacggtggagcagtgctcacggtgacgccggctcacctggttagcgtttggcagccggagagccagaagctcacgtttgtgtttgcggatcgcatcgaggagaagaaccaggtgctcgtacgggatgtggagacgggcgagctgaggccccagcgagtcgtcaaggtgggcagtgtgcgcagtaagggcgtggtcgcgccgctgacccgcgagggcaccattgtggtcaactcggtggccgccagttgctatgcggtgatcaacagccagtcgctggcccactggggactggctcccatgcgcctgctgtccacgctggaggcgtggctgcccgccaaggagcagttgcacagttcgccgaaggtggtgagctcggcgcagcagcagaatggcatccattggtatgccaatgcgctctacaaggtcaaggactacgttctgccgcagagctggcgccacgat

MDNHSSVPWASAASVTCLSLDAKCHSSSSSSSSKSAASSISAIPQEETQTMRHIAHTQRCLSRLTSLVALLLIVLPMVFSPAHSSRARNLYPLVLKQTIPNLSEYTNSASGPLEGVIRRDSPKFKDLVPNYNRDILFRDEEGTGADRLMSKRCKEKLNVLAYSVMNEWPGIRLLVTESWDEDYHHGQESLHYEGRAVTIATSDRDQSKYGMLARLAVEAGFDWVSYVSRRHIYCSVKSDSSISSHVHGCFTPESTALLESGVRKPLGELSIGDRVLSMTANGQAVYSEVILFMDRNLEQMQNFVQLHTDGGAVLTVTPAHLVSVWQPESQKLTFVFADRIEEKNQVLVRDVETGELRPQRVVKVGSVRSKGVVAPLTREGTIVVNSVAASCYAVINSQSLAHWGLAPMRLLSTLEAWLPAKEQLHSSPKVVSSAQQQNGIHWYANALYKVKDYVLPQSWRHD

Hh^C85S;Δ86-94^

atggataaccacagctcagtgccttgggccagtgccgccagtgtcacctgtctctccctggatgccaaatgccacagttccagttccagttccagctccaaatccgcagcgagctccatctccgcaatcccgcaagaagaaacgcaaacgatgcgccacattgcgcatacgcagcgttgcctcagcaggctgacctctctggtggccctgctgctgatcgtcttgccgatggtctttagcccggctcacagcagcgcgcgcaacctgtatccgctggtcctcaagcagacaattcccaatctatccgagtacacgaacagcgcctccggacctctggagggtgtgatccgtcgggactcgcccaaattcaaggacctcgtgcccaactacaacagggacatccttttccgcgacgaggaaggcaccggagcggatcgcttgatgagcaagcgctgcaaggagaagctaaacgtgctggcctactcggtgatgaacgaatggcccggcatccggctgctggtcaccgagagctgggacgaggactaccatcacggccaggagtcgctccactacgagggccgagcggtgaccattgccacctccgatcgcgaccagtccaaatacggcatgctcgctcgcctggccgtcgaggctggattcgattgggtctcctacgtcagcaggcgccacatctactgctccgtcaagtcagattcgtcgatcagttcccacgtgcacggctgcttcacgccggagagcacagcgctgctggagagtggagtccggaagccgctcggcgagctctctatcggagatcgtgttttgagcatgaccgccaacggacaggccgtctacagcgaagtgatcctcttcatggaccgcaacctcgagcagatgcaaaactttgtgcagctgcacacggacggtggagcagtgctcacggtgacgccggctcacctggttagcgtttggcagccggagagccagaagctcacgtttgtgtttgcggatcgcatcgaggagaagaaccaggtgctcgtacgggatgtggagacgggcgagctgaggccccagcgagtcgtcaaggtgggcagtgtgcgcagtaagggcgtggtcgcgccgctgacccgcgagggcaccattgtggtcaactcggtggccgccagttgctatgcggtgatcaacagccagtcgctggcccactggggactggctcccatgcgcctgctgtccacgctggaggcgtggctgcccgccaaggagcagttgcacagttcgccgaaggtggtgagctcggcgcagcagcagaatggcatccattggtatgccaatgcgctctacaaggtcaaggactacgttctgccgcagagctggcgccacgat

MDNHSSVPWASAASVTCLSLDAKCHSSSSSSSSKSAASSISAIPQEETQTMRHIAHTQRCLSRLTSLVALLLIVLPMVFSPAHSSARNLYPLVLKQTIPNLSEYTNSASGPLEGVIRRDSPKFKDLVPNYNRDILFRDEEGTGADRLMSKRCKEKLNVLAYSVMNEWPGIRLLVTESWDEDYHHGQESLHYEGRAVTIATSDRDQSKYGMLARLAVEAGFDWVSYVSRRHIYCSVKSDSSISSHVHGCFTPESTALLESGVRKPLGELSIGDRVLSMTANGQAVYSEVILFMDRNLEQMQNFVQLHTDGGAVLTVTPAHLVSVWQPESQKLTFVFADRIEEKNQVLVRDVETGELRPQRVVKVGSVRSKGVVAPLTREGTIVVNSVAASCYAVINSQSLAHWGLAPMRLLSTLEAWLPAKEQLHSSPKVVSSAQQQNGIHWYANALYKVKDYVLPQSWRHD

Hh^C85S;Δ86-95^

atggataaccacagctcagtgccttgggccagtgccgccagtgtcacctgtctctccctggatgccaaatgccacagttccagttccagttccagctccaaatccgcagcgagctccatctccgcaatcccgcaagaagaaacgcaaacgatgcgccacattgcgcatacgcagcgttgcctcagcaggctgacctctctggtggccctgctgctgatcgtcttgccgatggtctttagcccggctcacagcagccgcaacctgtatccgctggtcctcaagcagacaattcccaatctatccgagtacacgaacagcgcctccggacctctggagggtgtgatccgtcgggactcgcccaaattcaaggacctcgtgcccaactacaacagggacatccttttccgcgacgaggaaggcaccggagcggatcgcttgatgagcaagcgctgcaaggagaagctaaacgtgctggcctactcggtgatgaacgaatggcccggcatccggctgctggtcaccgagagctgggacgaggactaccatcacggccaggagtcgctccactacgagggccgagcggtgaccattgccacctccgatcgcgaccagtccaaatacggcatgctcgctcgcctggccgtcgaggctggattcgattgggtctcctacgtcagcaggcgccacatctactgctccgtcaagtcagattcgtcgatcagttcccacgtgcacggctgcttcacgccggagagcacagcgctgctggagagtggagtccggaagccgctcggcgagctctctatcggagatcgtgttttgagcatgaccgccaacggacaggccgtctacagcgaagtgatcctcttcatggaccgcaacctcgagcagatgcaaaactttgtgcagctgcacacggacggtggagcagtgctcacggtgacgccggctcacctggttagcgtttggcagccggagagccagaagctcacgtttgtgtttgcggatcgcatcgaggagaagaaccaggtgctcgtacgggatgtggagacgggcgagctgaggccccagcgagtcgtcaaggtgggcagtgtgcgcagtaagggcgtggtcgcgccgctgacccgcgagggcaccattgtggtcaactcggtggccgccagttgctatgcggtgatcaacagccagtcgctggcccactggggactggctcccatgcgcctgctgtccacgctggaggcgtggctgcccgccaaggagcagttgcacagttcgccgaaggtggtgagctcggcgcagcagcagaatggcatccattggtatgccaatgcgctctacaaggtcaaggactacgttctgccgcagagctggcgccacgat

MDNHSSVPWASAASVTCLSLDAKCHSSSSSSSSKSAASSISAIPQEETQTMRHIAHTQRCLSRLTSLVALLLIVLPMVFSPAHSSRNLYPLVLKQTIPNLSEYTNSASGPLEGVIRRDSPKFKDLVPNYNRDILFRDEEGTGADRLMSKRCKEKLNVLAYSVMNEWPGIRLLVTESWDEDYHHGQESLHYEGRAVTIATSDRDQSKYGMLARLAVEAGFDWVSYVSRRHIYCSVKSDSSISSHVHGCFTPESTALLESGVRKPLGELSIGDRVLSMTANGQAVYSEVILFMDRNLEQMQNFVQLHTDGGAVLTVTPAHLVSVWQPESQKLTFVFADRIEEKNQVLVRDVETGELRPQRVVKVGSVRSKGVVAPLTREGTIVVNSVAASCYAVINSQSLAHWGLAPMRLLSTLEAWLPAKEQLHSSPKVVSSAQQQNGIHWYANALYKVKDYVLPQSWRHD

Hh^C85S;Δ86-96^

atggataaccacagctcagtgccttgggccagtgccgccagtgtcacctgtctctccctggatgccaaatgccacagttccagttccagttccagctccaaatccgcagcgagctccatctccgcaatcccgcaagaagaaacgcaaacgatgcgccacattgcgcatacgcagcgttgcctcagcaggctgacctctctggtggccctgctgctgatcgtcttgccgatggtctttagcccggctcacagcagcaacctgtatccgctggtcctcaagcagacaattcccaatctatccgagtacacgaacagcgcctccggacctctggagggtgtgatccgtcgggactcgcccaaattcaaggacctcgtgcccaactacaacagggacatccttttccgcgacgaggaaggcaccggagcggatcgcttgatgagcaagcgctgcaaggagaagctaaacgtgctggcctactcggtgatgaacgaatggcccggcatccggctgctggtcaccgagagctgggacgaggactaccatcacggccaggagtcgctccactacgagggccgagcggtgaccattgccacctccgatcgcgaccagtccaaatacggcatgctcgctcgcctggccgtcgaggctggattcgattgggtctcctacgtcagcaggcgccacatctactgctccgtcaagtcagattcgtcgatcagttcccacgtgcacggctgcttcacgccggagagcacagcgctgctggagagtggagtccggaagccgctcggcgagctctctatcggagatcgtgttttgagcatgaccgccaacggacaggccgtctacagcgaagtgatcctcttcatggaccgcaacctcgagcagatgcaaaactttgtgcagctgcacacggacggtggagcagtgctcacggtgacgccggctcacctggttagcgtttggcagccggagagccagaagctcacgtttgtgtttgcggatcgcatcgaggagaagaaccaggtgctcgtacgggatgtggagacgggcgagctgaggccccagcgagtcgtcaaggtgggcagtgtgcgcagtaagggcgtggtcgcgccgctgacccgcgagggcaccattgtggtcaactcggtggccgccagttgctatgcggtgatcaacagccagtcgctggcccactggggactggctcccatgcgcctgctgtccacgctggaggcgtggctgcccgccaaggagcagttgcacagttcgccgaaggtggtgagctcggcgcagcagcagaatggcatccattggtatgccaatgcgctctacaaggtcaaggactacgttctgccgcagagctggcgccacgat

MDNHSSVPWASAASVTCLSLDAKCHSSSSSSSSKSAASSISAIPQEETQTMRHIAHTQRCLSRLTSLVALLLIVLPMVFSPAHSSNLYPLVLKQTIPNLSEYTNSASGPLEGVIRRDSPKFKDLVPNYNRDILFRDEEGTGADRLMSKRCKEKLNVLAYSVMNEWPGIRLLVTESWDEDYHHGQESLHYEGRAVTIATSDRDQSKYGMLARLAVEAGFDWVSYVSRRHIYCSVKSDSSISSHVHGCFTPESTALLESGVRKPLGELSIGDRVLSMTANGQAVYSEVILFMDRNLEQMQNFVQLHTDGGAVLTVTPAHLVSVWQPESQKLTFVFADRIEEKNQVLVRDVETGELRPQRVVKVGSVRSKGVVAPLTREGTIVVNSVAASCYAVINSQSLAHWGLAPMRLLSTLEAWLPAKEQLHSSPKVVSSAQQQNGIHWYANALYKVKDYVLPQSWRHD

Hh^C85S;Δ86-97^

atggataaccacagctcagtgccttgggccagtgccgccagtgtcacctgtctctccctggatgccaaatgccacagttccagttccagttccagctccaaatccgcagcgagctccatctccgcaatcccgcaagaagaaacgcaaacgatgcgccacattgcgcatacgcagcgttgcctcagcaggctgacctctctggtggccctgctgctgatcgtcttgccgatggtctttagcccggctcacagcagcctgtatccgctggtcctcaagcagacaattcccaatctatccgagtacacgaacagcgcctccggacctctggagggtgtgatccgtcgggactcgcccaaattcaaggacctcgtgcccaactacaacagggacatccttttccgcgacgaggaaggcaccggagcggatcgcttgatgagcaagcgctgcaaggagaagctaaacgtgctggcctactcggtgatgaacgaatggcccggcatccggctgctggtcaccgagagctgggacgaggactaccatcacggccaggagtcgctccactacgagggccgagcggtgaccattgccacctccgatcgcgaccagtccaaatacggcatgctcgctcgcctggccgtcgaggctggattcgattgggtctcctacgtcagcaggcgccacatctactgctccgtcaagtcagattcgtcgatcagttcccacgtgcacggctgcttcacgccggagagcacagcgctgctggagagtggagtccggaagccgctcggcgagctctctatcggagatcgtgttttgagcatgaccgccaacggacaggccgtctacagcgaagtgatcctcttcatggaccgcaacctcgagcagatgcaaaactttgtgcagctgcacacggacggtggagcagtgctcacggtgacgccggctcacctggttagcgtttggcagccggagagccagaagctcacgtttgtgtttgcggatcgcatcgaggagaagaaccaggtgctcgtacgggatgtggagacgggcgagctgaggccccagcgagtcgtcaaggtgggcagtgtgcgcagtaagggcgtggtcgcgccgctgacccgcgagggcaccattgtggtcaactcggtggccgccagttgctatgcggtgatcaacagccagtcgctggcccactggggactggctcccatgcgcctgctgtccacgctggaggcgtggctgcccgccaaggagcagttgcacagttcgccgaaggtggtgagctcggcgcagcagcagaatggcatccattggtatgccaatgcgctctacaaggtcaaggactacgttctgccgcagagctggcgccacgat

MDNHSSVPWASAASVTCLSLDAKCHSSSSSSSSKSAASSISAIPQEETQTMRHIAHTQRCLSRLTSLVALLLIVLPMVFSPAHSSLYPLVLKQTIPNLSEYTNSASGPLEGVIRRDSPKFKDLVPNYNRDILFRDEEGTGADRLMSKRCKEKLNVLAYSVMNEWPGIRLLVTESWDEDYHHGQESLHYEGRAVTIATSDRDQSKYGMLARLAVEAGFDWVSYVSRRHIYCSVKSDSSISSHVHGCFTPESTALLESGVRKPLGELSIGDRVLSMTANGQAVYSEVILFMDRNLEQMQNFVQLHTDGGAVLTVTPAHLVSVWQPESQKLTFVFADRIEEKNQVLVRDVETGELRPQRVVKVGSVRSKGVVAPLTREGTIVVNSVAASCYAVINSQSLAHWGLAPMRLLSTLEAWLPAKEQLHSSPKVVSSAQQQNGIHWYANALYKVKDYVLPQSWRHD

Hh^C85S;Δ86-98^

atggataaccacagctcagtgccttgggccagtgccgccagtgtcacctgtctctccctggatgccaaatgccacagttccagttccagttccagctccaaatccgcagcgagctccatctccgcaatcccgcaagaagaaacgcaaacgatgcgccacattgcgcatacgcagcgttgcctcagcaggctgacctctctggtggccctgctgctgatcgtcttgccgatggtctttagcccggctcacagcagctatccgctggtcctcaagcagacaattcccaatctatccgagtacacgaacagcgcctccggacctctggagggtgtgatccgtcgggactcgcccaaattcaaggacctcgtgcccaactacaacagggacatccttttccgcgacgaggaaggcaccggagcggatcgcttgatgagcaagcgctgcaaggagaagctaaacgtgctggcctactcggtgatgaacgaatggcccggcatccggctgctggtcaccgagagctgggacgaggactaccatcacggccaggagtcgctccactacgagggccgagcggtgaccattgccacctccgatcgcgaccagtccaaatacggcatgctcgctcgcctggccgtcgaggctggattcgattgggtctcctacgtcagcaggcgccacatctactgctccgtcaagtcagattcgtcgatcagttcccacgtgcacggctgcttcacgccggagagcacagcgctgctggagagtggagtccggaagccgctcggcgagctctctatcggagatcgtgttttgagcatgaccgccaacggacaggccgtctacagcgaagtgatcctcttcatggaccgcaacctcgagcagatgcaaaactttgtgcagctgcacacggacggtggagcagtgctcacggtgacgccggctcacctggttagcgtttggcagccggagagccagaagctcacgtttgtgtttgcggatcgcatcgaggagaagaaccaggtgctcgtacgggatgtggagacgggcgagctgaggccccagcgagtcgtcaaggtgggcagtgtgcgcagtaagggcgtggtcgcgccgctgacccgcgagggcaccattgtggtcaactcggtggccgccagttgctatgcggtgatcaacagccagtcgctggcccactggggactggctcccatgcgcctgctgtccacgctggaggcgtggctgcccgccaaggagcagttgcacagttcgccgaaggtggtgagctcggcgcagcagcagaatggcatccattggtatgccaatgcgctctacaaggtcaaggactacgttctgccgcagagctggcgccacgat

MDNHSSVPWASAASVTCLSLDAKCHSSSSSSSSKSAASSISAIPQEETQTMRHIAHTQRCLSRLTSLVALLLIVLPMVFSPAHSSYPLVLKQTIPNLSEYTNSASGPLEGVIRRDSPKFKDLVPNYNRDILFRDEEGTGADRLMSKRCKEKLNVLAYSVMNEWPGIRLLVTESWDEDYHHGQESLHYEGRAVTIATSDRDQSKYGMLARLAVEAGFDWVSYVSRRHIYCSVKSDSSISSHVHGCFTPESTALLESGVRKPLGELSIGDRVLSMTANGQAVYSEVILFMDRNLEQMQNFVQLHTDGGAVLTVTPAHLVSVWQPESQKLTFVFADRIEEKNQVLVRDVETGELRPQRVVKVGSVRSKGVVAPLTREGTIVVNSVAASCYAVINSQSLAHWGLAPMRLLSTLEAWLPAKEQLHSSPKVVSSAQQQNGIHWYANALYKVKDYVLPQSWRHD

Hh^C85S;Δ86-99^

atggataaccacagctcagtgccttgggccagtgccgccagtgtcacctgtctctccctggatgccaaatgccacagttccagttccagttccagctccaaatccgcagcgagctccatctccgcaatcccgcaagaagaaacgcaaacgatgcgccacattgcgcatacgcagcgttgcctcagcaggctgacctctctggtggccctgctgctgatcgtcttgccgatggtctttagcccggctcacagcagcccgctggtcctcaagcagacaattcccaatctatccgagtacacgaacagcgcctccggacctctggagggtgtgatccgtcgggactcgcccaaattcaaggacctcgtgcccaactacaacagggacatccttttccgcgacgaggaaggcaccggagcggatcgcttgatgagcaagcgctgcaaggagaagctaaacgtgctggcctactcggtgatgaacgaatggcccggcatccggctgctggtcaccgagagctgggacgaggactaccatcacggccaggagtcgctccactacgagggccgagcggtgaccattgccacctccgatcgcgaccagtccaaatacggcatgctcgctcgcctggccgtcgaggctggattcgattgggtctcctacgtcagcaggcgccacatctactgctccgtcaagtcagattcgtcgatcagttcccacgtgcacggctgcttcacgccggagagcacagcgctgctggagagtggagtccggaagccgctcggcgagctctctatcggagatcgtgttttgagcatgaccgccaacggacaggccgtctacagcgaagtgatcctcttcatggaccgcaacctcgagcagatgcaaaactttgtgcagctgcacacggacggtggagcagtgctcacggtgacgccggctcacctggttagcgtttggcagccggagagccagaagctcacgtttgtgtttgcggatcgcatcgaggagaagaaccaggtgctcgtacgggatgtggagacgggcgagctgaggccccagcgagtcgtcaaggtgggcagtgtgcgcagtaagggcgtggtcgcgccgctgacccgcgagggcaccattgtggtcaactcggtggccgccagttgctatgcggtgatcaacagccagtcgctggcccactggggactggctcccatgcgcctgctgtccacgctggaggcgtggctgcccgccaaggagcagttgcacagttcgccgaaggtggtgagctcggcgcagcagcagaatggcatccattggtatgccaatgcgctctacaaggtcaaggactacgttctgccgcagagctggcgccacgat

MDNHSSVPWASAASVTCLSLDAKCHSSSSSSSSKSAASSISAIPQEETQTMRHIAHTQRCLSRLTSLVALLLIVLPMVFSPAHSSPLVLKQTIPNLSEYTNSASGPLEGVIRRDSPKFKDLVPNYNRDILFRDEEGTGADRLMSKRCKEKLNVLAYSVMNEWPGIRLLVTESWDEDYHHGQESLHYEGRAVTIATSDRDQSKYGMLARLAVEAGFDWVSYVSRRHIYCSVKSDSSISSHVHGCFTPESTALLESGVRKPLGELSIGDRVLSMTANGQAVYSEVILFMDRNLEQMQNFVQLHTDGGAVLTVTPAHLVSVWQPESQKLTFVFADRIEEKNQVLVRDVETGELRPQRVVKVGSVRSKGVVAPLTREGTIVVNSVAASCYAVINSQSLAHWGLAPMRLLSTLEAWLPAKEQLHSSPKVVSSAQQQNGIHWYANALYKVKDYVLPQSWRHD

Hh^C85S;Δ86-100^

atggataaccacagctcagtgccttgggccagtgccgccagtgtcacctgtctctccctggatgccaaatgccacagttccagttccagttccagctccaaatccgcagcgagctccatctccgcaatcccgcaagaagaaacgcaaacgatgcgccacattgcgcatacgcagcgttgcctcagcaggctgacctctctggtggccctgctgctgatcgtcttgccgatggtctttagcccggctcacagcagcctggtcctcaagcagacaattcccaatctatccgagtacacgaacagcgcctccggacctctggagggtgtgatccgtcgggactcgcccaaattcaaggacctcgtgcccaactacaacagggacatccttttccgcgacgaggaaggcaccggagcggatcgcttgatgagcaagcgctgcaaggagaagctaaacgtgctggcctactcggtgatgaacgaatggcccggcatccggctgctggtcaccgagagctgggacgaggactaccatcacggccaggagtcgctccactacgagggccgagcggtgaccattgccacctccgatcgcgaccagtccaaatacggcatgctcgctcgcctggccgtcgaggctggattcgattgggtctcctacgtcagcaggcgccacatctactgctccgtcaagtcagattcgtcgatcagttcccacgtgcacggctgcttcacgccggagagcacagcgctgctggagagtggagtccggaagccgctcggcgagctctctatcggagatcgtgttttgagcatgaccgccaacggacaggccgtctacagcgaagtgatcctcttcatggaccgcaacctcgagcagatgcaaaactttgtgcagctgcacacggacggtggagcagtgctcacggtgacgccggctcacctggttagcgtttggcagccggagagccagaagctcacgtttgtgtttgcggatcgcatcgaggagaagaaccaggtgctcgtacgggatgtggagacgggcgagctgaggccccagcgagtcgtcaaggtgggcagtgtgcgcagtaagggcgtggtcgcgccgctgacccgcgagggcaccattgtggtcaactcggtggccgccagttgctatgcggtgatcaacagccagtcgctggcccactggggactggctcccatgcgcctgctgtccacgctggaggcgtggctgcccgccaaggagcagttgcacagttcgccgaaggtggtgagctcggcgcagcagcagaatggcatccattggtatgccaatgcgctctacaaggtcaaggactacgttctgccgcagagctggcgccacgat

MDNHSSVPWASAASVTCLSLDAKCHSSSSSSSSKSAASSISAIPQEETQTMRHIAHTQRCLSRLTSLVALLLIVLPMVFSPAHSSLVLKQTIPNLSEYTNSASGPLEGVIRRDSPKFKDLVPNYNRDILFRDEEGTGADRLMSKRCKEKLNVLAYSVMNEWPGIRLLVTESWDEDYHHGQESLHYEGRAVTIATSDRDQSKYGMLARLAVEAGFDWVSYVSRRHIYCSVKSDSSISSHVHGCFTPESTALLESGVRKPLGELSIGDRVLSMTANGQAVYSEVILFMDRNLEQMQNFVQLHTDGGAVLTVTPAHLVSVWQPESQKLTFVFADRIEEKNQVLVRDVETGELRPQRVVKVGSVRSKGVVAPLTREGTIVVNSVAASCYAVINSQSLAHWGLAPMRLLSTLEAWLPAKEQLHSSPKVVSSAQQQNGIHWYANALYKVKDYVLPQSWRHD

^HA^Hh

atggataaccacagctcagtgccttgggccagtgccgccagtgtcacctgtctctccctggatgccaaatgccacagttccagttccagttccagctccaaatccgcagcgagctccatctccgcaatcccgcaagaagaaacgcaaacgatgcgccacattgcgcatacgcagcgttgcctcagcaggctgacctctctggtggccctgctgctgatcgtcttgccgatggtctttagcccggctcacagctgcggtcctggccgaggattgTACCCATACGATGTTCCAGATTACGCTggtcgtcatagggcgcgcaacctgtatccgctggtcctcaagcagacaattcccaatctatccgagtacacgaacagcgcctccggacctctggagggtgtgatccgtcgggactcgcccaaattcaaggacctcgtgcccaactacaacagggacatccttttccgcgacgaggaaggcaccggagcggatcgcttgatgagcaagcgctgcaaggagaagctaaacgtgctggcctactcggtgatgaacgaatggcccggcatccggctgctggtcaccgagagctgggacgaggactaccatcacggccaggagtcgctccactacgagggccgagcggtgaccattgccacctccgatcgcgaccagtccaaatacggcatgctcgctcgcctggccgtcgaggctggattcgattgggtctcctacgtcagcaggcgccacatctactgctccgtcaagtcagattcgtcgatcagttcccacgtgcacggctgcttcacgccggagagcacagcgctgctggagagtggagtccggaagccgctcggcgagctctctatcggagatcgtgttttgagcatgaccgccaacggacaggccgtctacagcgaagtgatcctcttcatggaccgcaacctcgagcagatgcaaaactttgtgcagctgcacacggacggtggagcagtgctcacggtgacgccggctcacctggttagcgtttggcagccggagagccagaagctcacgtttgtgtttgcggatcgcatcgaggagaagaaccaggtgctcgtacgggatgtggagacgggcgagctgaggccccagcgagtcgtcaaggtgggcagtgtgcgcagtaagggcgtggtcgcgccgctgacccgcgagggcaccattgtggtcaactcggtggccgccagttgctatgcggtgatcaacagccagtcgctggcccactggggactggctcccatgcgcctgctgtccacgctggaggcgtggctgcccgccaaggagcagttgcacagttcgccgaaggtggtgagctcggcgcagcagcagaatggcatccattggtatgccaatgcgctctacaaggtcaaggactacgttctgccgcagagctggcgccacgat

MDNHSSVPWASAASVTCLSLDAKCHSSSSSSSSKSAASSISAIPQEETQTMRHIAHTQRCLSRLTSLVALLLIVLPMVFSPAHSCGPGRGLYPYDVPDYAGRHRARNLYPLVLKQTIPNLSEYTNSASGPLEGVIRRDSPKFKDLVPNYNRDILFRDEEGTGADRLMSKRCKEKLNVLAYSVMNEWPGIRLLVTESWDEDYHHGQESLHYEGRAVTIATSDRDQSKYGMLARLAVEAGFDWVSYVSRRHIYCSVKSDSSISSHVHGCFTPESTALLESGVRKPLGELSIGDRVLSMTANGQAVYSEVILFMDRNLEQMQNFVQLHTDGGAVLTVTPAHLVSVWQPESQKLTFVFADRIEEKNQVLVRDVETGELRPQRVVKVGSVRSKGVVAPLTREGTIVVNSVAASCYAVINSQSLAHWGLAPMRLLSTLEAWLPAKEQLHSSPKVVSSAQQQNGIHWYANALYKVKDYVLPQSWRHD

^HA^Hh^C85S^

atggataaccacagctcagtgccttgggccagtgccgccagtgtcacctgtctctccctggatgccaaatgccacagttccagttccagttccagctccaaatccgcagcgagctccatctccgcaatcccgcaagaagaaacgcaaacgatgcgccacattgcgcatacgcagcgttgcctcagcaggctgacctctctggtggccctgctgctgatcgtcttgccgatggtctttagcccggctcacagcagcggtcctggccgaggattgTACCCATACGATGTTCCAGATTACGCTggtcgtcatagggcgcgcaacctgtatccgctggtcctcaagcagacaattcccaatctatccgagtacacgaacagcgcctccggacctctggagggtgtgatccgtcgggactcgcccaaattcaaggacctcgtgcccaactacaacagggacatccttttccgcgacgaggaaggcaccggagcggatcgcttgatgagcaagcgctgcaaggagaagctaaacgtgctggcctactcggtgatgaacgaatggcccggcatccggctgctggtcaccgagagctgggacgaggactaccatcacggccaggagtcgctccactacgagggccgagcggtgaccattgccacctccgatcgcgaccagtccaaatacggcatgctcgctcgcctggccgtcgaggctggattcgattgggtctcctacgtcagcaggcgccacatctactgctccgtcaagtcagattcgtcgatcagttcccacgtgcacggctgcttcacgccggagagcacagcgctgctggagagtggagtccggaagccgctcggcgagctctctatcggagatcgtgttttgagcatgaccgccaacggacaggccgtctacagcgaagtgatcctcttcatggaccgcaacctcgagcagatgcaaaactttgtgcagctgcacacggacggtggagcagtgctcacggtgacgccggctcacctggttagcgtttggcagccggagagccagaagctcacgtttgtgtttgcggatcgcatcgaggagaagaaccaggtgctcgtacgggatgtggagacgggcgagctgaggccccagcgagtcgtcaaggtgggcagtgtgcgcagtaagggcgtggtcgcgccgctgacccgcgagggcaccattgtggtcaactcggtggccgccagttgctatgcggtgatcaacagccagtcgctggcccactggggactggctcccatgcgcctgctgtccacgctggaggcgtggctgcccgccaaggagcagttgcacagttcgccgaaggtggtgagctcggcgcagcagcagaatggcatccattggtatgccaatgcgctctacaaggtcaaggactacgttctgccgcagagctggcgccacgat

MDNHSSVPWASAASVTCLSLDAKCHSSSSSSSSKSAASSISAIPQEETQTMRHIAHTQRCLSRLTSLVALLLIVLPMVFSPAHSSGPGRGLYPYDVPDYAGRHRARNLYPLVLKQTIPNLSEYTNSASGPLEGVIRRDSPKFKDLVPNYNRDILFRDEEGTGADRLMSKRCKEKLNVLAYSVMNEWPGIRLLVTESWDEDYHHGQESLHYEGRAVTIATSDRDQSKYGMLARLAVEAGFDWVSYVSRRHIYCSVKSDSSISSHVHGCFTPESTALLESGVRKPLGELSIGDRVLSMTANGQAVYSEVILFMDRNLEQMQNFVQLHTDGGAVLTVTPAHLVSVWQPESQKLTFVFADRIEEKNQVLVRDVETGELRPQRVVKVGSVRSKGVVAPLTREGTIVVNSVAASCYAVINSQSLAHWGLAPMRLLSTLEAWLPAKEQLHSSPKVVSSAQQQNGIHWYANALYKVKDYVLPQSWRHD
